# Rare Histological Variants of Liver Cancer and Their Management: A Single-Institution Experience

**DOI:** 10.1155/2021/6654229

**Published:** 2021-04-21

**Authors:** Brandon Swed, Omar Gandarilla, Kenrry Chiu, Karim H. Halazun, Benjamin Samstein, Rhonda Yantiss, Gagandeep Brar

**Affiliations:** ^1^Division of Hematology and Medical Oncology, Department of Medicine, Weill Cornell Medicine/New-York Presbyterian, New York, New York, USA; ^2^Department of Pathology and Laboratory Medicine, Weill Cornell Medicine/New-York Presbyterian, New York, New York, USA; ^3^Division of Liver Transplantation and Hepatobiliary Surgery, Department of Surgery, Weill Cornell Medicine/New-York Presbyterian, New York, New York, USA

## Abstract

Primary liver malignancies, including hepatocellular carcinoma (HCC) and cholangiocarcinoma, are a major cause of cancer-related morbidity and mortality worldwide. There are several histologically and biologically distinct subtypes of liver cancer that have previously been reported. However, literature regarding the nonsurgical management of these patients upon disease recurrence remains limited. These variants include combined HCC-cholangiocarcinoma (cHCC-CC), Epstein–Barr virus- (EBV-) associated carcinoma, undifferentiated carcinoma, and clear cell or thyroid-like variants of HCC. Here, we aim to highlight the pathologic features, clinical course, and outcomes of five patients with these unusual hepatic tumors and explain the rationale behind the choice of their systemic therapies upon disease recurrence. All patients underwent surgical resection as the standard of care for localized disease, and upon relapse, they were treated with either chemotherapy, targeted therapy, immunotherapy, or active surveillance based on the clinical context and tumor histology. These rare variants are important to recognize as they have prognostic and therapeutic implications, and there are currently insufficient data in the literature to guide further therapy.

## 1. Introduction

Primary liver cancers are a major cause of morbidity and mortality worldwide. Hepatocellular carcinoma (HCC), the most common type of liver cancer in adults, is ranked as the sixth most prevalent solid tumor malignancy and the third leading cause of cancer-related deaths globally [1]. Despite several recent advances in diagnostic and therapeutic modalities, prognosis generally remains poor, with a five-year relative survival rate of 18% observed in the United States [2]. Hepatic resection and liver transplantation are the standard of care for localized disease and are among the only treatment options with curative intent, as conventional chemotherapy and radiation therapy are generally ineffective [3]. Cholangiocarcinoma, the next most common primary liver cancer, is also associated with a high mortality that is generally attributed to advanced stage disease at the time of diagnosis. Several prognostic factors for liver cancers have been proposed, including tumor size, vascular invasion, elevated serum tumor markers (e.g., alpha-fetoprotein (AFP) and carbohydrate antigen 19-9 (CA 19-9)), resection margin status, and tumor histology [4].

Histologic analysis remains the gold standard for the diagnosis and classification of primary liver carcinomas, although the diagnosis of HCC can usually be made radiographically in the appropriate clinical context, and thus, many tumors are treated prior to histologic evaluation. However, there are some relatively rare variants that may be important to recognize because they either have prognostic and therapeutic implications or simulate features of other malignancies [5, 6]. These include combined HCC-cholangiocarcinoma (cHCC-CC), Epstein–Barr virus- (EBV-) associated carcinoma, undifferentiated carcinoma, and clear cell or thyroid-like variants of HCC. Here, we report on the clinical course and outcomes of five patients with unusual hepatic tumors and describe their clinical and pathologic features.

## 2. Case Reports

### 2.1. Case 1: cHCC/CCA

A 67-year-old Caucasian male with a history of combined hepatitis C and alcoholic cirrhosis was found to have a small hepatic lesion in segment V on a screening ultrasound in November 2019. Follow-up abdominal MRI revealed a 1.5 cm Liver Imaging Reporting and Data System (LI-RADS) 4 lesion which was again confirmed on PET/CT (SUV 7.8) without evidence of distant metastases.

**Figure 1:**
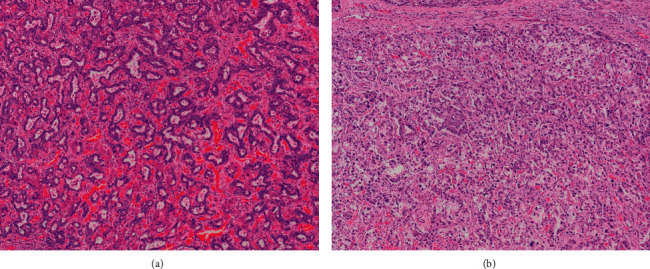
The tumour showing mixed morphologic features with haphazardly arranged, irregularly shaped glands enmeshed in fibrotic stroma typical of cholangiocarcinoma (a) as well as sheet-like growth of solid cell nests and trabeculae showing morphologic and immunohistochemical evidence of hepatocellular differentiation (b).

Given radiographic evidence of HCC and elevated AFP to 1017 ng/mL, he underwent a partial left hepatectomy the following month. The cirrhotic liver contained a 1.5 cm firm white tumor that showed morphologic and immunohistochemical features of a combined hepatocellular-cholangiocarcinoma (Figure 1). The adenocarcinomatous component showed CK7-positivity and was negative for arginase, whereas areas of hepatocellular differentiation were positive for arginase and negative for CK7. The tumor demonstrated extensive venous and lymphovascular invasion. Notably, his presurgical CA 19-9 was within normal limits.

Given pathology findings of positive margins and therefore high risk of recurrence, the decision was made to proceed with adjuvant stereotactic body radiation therapy (SBRT). His AFP declined to 9.8 ng/mL in the postoperative setting. He was started on systemic therapy with capecitabine in February 2020, with the intention of treating the more aggressive of the dual histologic subtypes, the cholangiocarcinoma. His AFP subsequently began to rise, and repeat imaging two months later unfortunately confirmed recurrence confined to the liver parenchyma. He had a repeat liver biopsy that demonstrated adenocarcinoma consistent with pancreaticobiliary origin. Further testing revealed PD-L1 positivity of 5% without targetable molecular alterations identified on next-generation Sequencing. He received local therapy with Y90 radioembolization and was started on nivolumab monotherapy which he is currently tolerating well. AFP has since downtrended.

### 2.2. Case 2: HCC/Undifferentiated Carcinoma

A 48-year-old Asian male was incidentally diagnosed with hepatitis B in June 2019 and started on antiviral therapy at that time. He developed focal right upper quadrant pain several months later prompting an abdominal ultrasound that revealed a region of heterogeneity in the right hepatic lobe, and follow-up abdominal MRI demonstrated a 14.7 × 10.1 cm LI-RADS TIV (tumor in vein) infiltrative hepatic mass consistent with HCC with extensive tumor thrombus. AFP level was elevated to 64.6 ng/mL, and staging CT scans and skeletal scintigraphy showed no evidence of distant metastatic disease.

**Figure 2:**
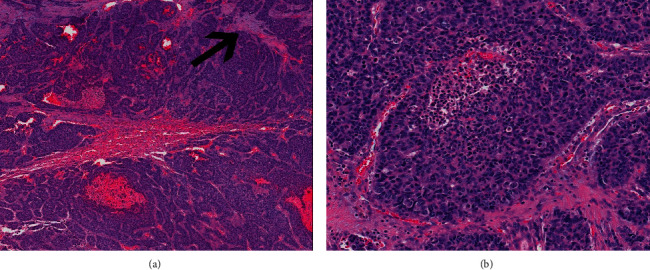
The tumour composed of variably sized nests and trabeculae associated with desmoplasia (arrow) and hemorrhage. (a) High-grade tumour cells containing enlarged, hyperchromatic nuclei with relatively scant cytoplasm extensive necrosis. (b) The high-grade areas are positive for cytokeratin-7 and show patchy staining for arginase and Hepar-1.

The patient underwent a right hepatectomy in December 2019 with complete resection of a 14.2 cm heterogeneous tumor accompanied by satellite nodules with extensive venous invasion. The carcinoma showed hepatocellular differentiation as well as undifferentiated components (Figure 2). The latter consisted of broad trabeculae and sheets of high-grade tumor cells with extensive necrosis and readily apparent mitotic figures. These areas lacked convincing staining for Hepar-1 and arginase but had stronger, more diffuse CK7 staining. The tumor was negative for synaptophysin, chromogranin, and CK19.

He had a repeat MRI two months postresection in the setting of recurrent abdominal pain and rising AFP that demonstrated diffuse infiltrative tumor in the left hepatic lobe and suspected right lower lobe pulmonary metastases. He was started on palliative chemotherapy, and after one cycle, succumbed to his disease.

### 2.3. Case 3: HCC, Clear Cell Variant

A 59-year-old Asian male with a history of hepatitis B diagnosed in 2011 on antiviral therapy since that time was noted to have an elevated AFP to 21.8 ng/mL and liver mass on screening abdominal ultrasound in March 2019. Abdominal MRI, which was delayed due to lack of short-term follow-up, showed a dominant 5.5 cm segment V LI-RADS M (probably or definitely malignant, not necessarily HCC) hepatic mass. Notably, on staging PET/CT, there was no evidence of FDG-avid disease in the kidneys, gastrointestinal tract, or other sites where clear cell carcinoma is known to originate.

**Figure 3:**
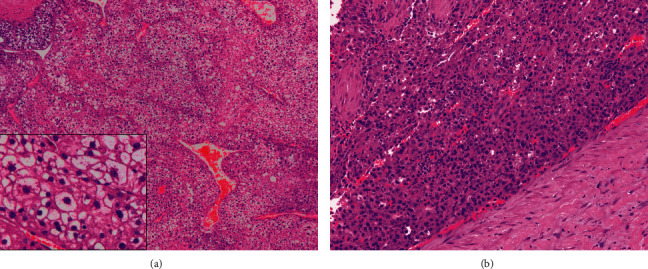
This hepatocellular carcinoma containing broad trabaculae of tumour cells with abundant clear cytoplasm (a). Other high-grade areas resembling conventional hepatocellular carcinoma (b).

He underwent a right partial hepatectomy in October 2019 with complete resection of a 6.1 cm variegated tumor surrounded by multiple satellite nodules. Portal vein invasion was identified. Most (>80%) of the tumor cells contained abundant clear cytoplasm with some higher grade areas (Figure 3). The background liver showed chronic hepatitis with mild activity and thin septal fibrosis consistent with regressed cirrhosis.

Postoperatively, he was noted to have a rising AFP to 154 ng/mL and underwent SBRT to the resection cavity. Repeat imaging in April 2020 showed multifocal progressive liver lesions with suspected pulmonary and osseous metastatic disease, and AFP rapidly increased to 2728 ng/mL. He was subsequently started on palliative systemic therapy with lenvatinib, followed by second-line nivolumab for further disease progression.

### 2.4. Case 4: CCA, Thyroid-Like Variant

A 60-year-old Caucasian female with a history of Hashimoto's thyroiditis was incidentally noted to have a liver mass on CT imaging in 2009 performed as part of a workup for abdominal pain. Follow-up MRI demonstrated radiographic findings consistent with a hepatic adenoma. On surveillance imaging, this lesion was noted to be enlarging in size with associated hemorrhage, and she ultimately underwent a left hepatectomy ten years later in October 2019.

**Figure 4:**
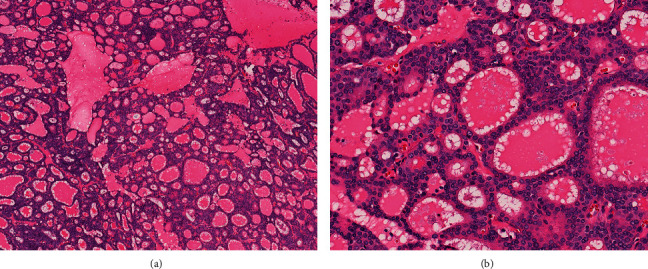
Sheets of tumour cells arranged in cystically dilated glands (a) that contain brightly eosinophilic secretions reminiscent of colloid (b).

The resection specimen contained an 11.0 cm tan, variegated hemorrhagic tumor with cystic degeneration arising in a noncirrhotic liver. Resection margins were negative, and no lymphovascular invasion was identified. Sheets of tumor cells were arranged in large, cystically dilated glands filled with eosinophilic material reminiscent of colloid seen in thyroid follicles (Figure 4). The entirety of the tumor demonstrated low-grade nuclear features without evidence of biliary dysplasia in the nonneoplastic parenchyma. The tumor cells were CK-7-positive and showed patchy synaptophysin staining, but were negative for arginase, TTF-1, and chromogranin. The tumor was classified as a thyroid-like variant of an intrahepatic cholangiocarcinoma.

A subsequent neck ultrasound showed no focal abnormalities to suggest metastatic disease from a primary thyroid malignancy. Given prior definitive surgical resection and the indolent nature of her neoplasm, the decision was made to defer adjuvant chemotherapy and instead pursue active radiographic surveillance. She has no evidence of recurrence nine months postresection.

### 2.5. Case 5: EBV-Associated CCA (Lymphoepithelioma-Like Carcinoma Variant of CCA)

A 28-year-old Caucasian female with a history of ulcerative colitis and primary sclerosing cholangitis diagnosed at ages 16 and 23, respectively, was found to have a 6 cm mass adjacent to the caudate lobe on a screening abdominal MRI in October 2017. Tumor markers were notable for an elevated CA 19-9 to 2,733 U/mL, while AFP and CEA were within normal limits. She underwent endoscopic biopsy of the subhepatic lesion, and fine needle aspiration (FNA) revealed adenocarcinoma, with a tumor profile that favored an upper gastrointestinal/pancreatobiliary primary. Staging PET/CT showed a peripancreatic/portacaval hypermetabolic mass likely representing the primary disease with suspected metastatic aortocaval lymphadenopathy but no evidence of distant metastatic disease. The following month, she had an excisional portal lymph node biopsy confirming metastatic adenocarcinoma.

**Figure 5:**
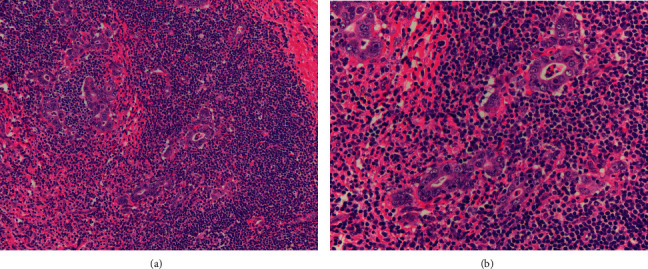
Infiltrating malignant glands are intimately associated with a dense lymphoid infiltrate (a). Intraepithelial lymphocytes are also present (b) ([7–12]).

She ultimately underwent a left hepatectomy with caudate lobectomy and hilar lymphadenectomy in March 2018. Surgical pathology revealed a 2.2 cm ill-defined, perihilar bile duct tumor centered at the confluence of the right and left hepatic ducts. The background liver showed features of primary sclerosing cholangitis with portal-based fibrosis. Microscopic examination of the tumor showed adenocarcinoma confined to the bile duct wall. The malignant glands were associated with a dense lymphoid infiltrate and tumor-infiltrating lymphocytes (Figure 5). In situ hybridization for EBV-encoded RNAs (EBER) demonstrated strong, diffuse labeling of tumor cells. One regional lymph node showed metastatic carcinoma. The findings were compatible with EBV-associated perihilar cholangiocarcinoma.

She experienced multiple postoperative complications including a hepatic abscess, severe gastroparesis, and malnutrition that precluded adjuvant systemic therapy. Her CA 19-9 normalized in the postoperative period, and surveillance scans to date have shown no evidence of recurrent disease.

## 3. Discussion

Despite its overall high morbidity and mortality, primary liver cancer is a disease with a remarkably variable clinical course. A timely and accurate diagnosis is crucial for optimal management of these patients, as they generally have a poor response to systemic therapy [3]. This is, at least in part, explained by the several histologically and biologically distinct subtypes of liver cancer that have been reported.

Such heterogeneity of clinical findings, imaging characteristics, and histopathologic features can often obscure the precise diagnosis in these patients. [4] For example, cHCC/CCA described in case 1 displays phenotypic features suggestive of both hepatic and biliary differentiation, making the preoperative diagnosis exceptionally challenging. This is true of the other tumor subtypes as well. As described in case 3, it is important to distinguish the atypical clear cell variant of HCC from metastatic clear cell carcinomas that arise from various other primary sites, including the kidneys, ovaries, lungs, and other organs [13, 14]. Similarly, the thyroid-like variant of intrahepatic cholangiocarcinoma discussed in case 4 can be misconstrued as metastatic disease or ectopic normal thyroid tissue. However, the absence of a primary thyroid neoplasm and a lack of thyroid markers like TTF-1 in the liver tumor can be useful in distinguishing these two entities [15, 16].

The histologically distinct tumor features highlighted in this case series have important prognostic and therapeutic implications. However, literature regarding nonsurgical management in these rare subtypes is scarce and mainly limited to case reports, as outlined in Table 1. We report five patients with unique variants of liver cancer, all of whom underwent surgical resection as the standard of care for localized disease. These are among some of the first reports of using systemic therapy to treat these rare variants. Although data to support this is lacking, one of the five patients (case 1, cHCC/CCA) received adjuvant systemic therapy based on his anticipated poor clinical trajectory in the context of positive resection margins and high risk of recurrence. His treatment was targeted toward the more aggressive cholangiocarcinoma subtype. [6] This is in contrast to the patient in case 4, for instance, with a slow-growing thyroid variant that appears to behave more like a low-grade carcinoma. The exceedingly rare prior reports of this subtype suggest predominance in young females with a late presentation and large tumor burden, without apparent underlying liver parenchymal disease. Two of the patients (case 2 with undifferentiated carcinoma and case 3 with clear cell variant) received systemic therapy at the time of disease recurrence or metastases, and all three of the patients who received systemic therapy had further progression of disease. Clear cell carcinoma of the liver has traditionally been associated with a more favorable outcome than conventional HCC. However, the patient presented in case 3 had an unusually aggressive course, possibly attributed to a subpopulation of cells with higher grade cytology.

As we learn more about the subtleties of these uncommon liver cancers, diagnostic and therapeutic dilemmas remain. Treatment for these patients has generally been directed toward their dominant histologic subtype, as there are insufficient data in the literature to guide further therapy. This case series highlights the need for a deeper understanding of the biology of these tumors in order to select the most appropriate treatment options to improve patient outcomes. Although no clinically useful genomic alterations were detected by next-generation sequencing assays performed for each of the patients described, it is important to recognize that correlation of histology with molecular data remains a powerful tool in the understanding of the pathogenesis and behavior of liver cancers. More studies are needed to explore this, as greater awareness of these distinct entities can lead to improved treatment strategies and patient outcomes.

## Figures and Tables

**Table 1:** Summary of selected prior case reports of liver cancer with rare histological features.

Reference	Patient demographics	Histological variant	HBV/HCV status	Tumor size (cm)	Solitary or multiple	Treatment	Vital status
Sakhuja et al. [13]	32, F	Clear cell	−/−	18	Solitary	Hepatic resection	Alive
Albores-Saavedra et al. [14]	63, M	Clear cell	−/−	0.9	Solitary	Hepatic resection	Alive
Albores-Saavedra et al. [14]	25, F	Clear cell	−/−	1.1	Solitary	Hepatic resection	Alive
Albores-Saavedra et al. [14]	64, M	Clear cell	−/−	6	Solitary	Hepatic resection	Alive
Pecorella et al. [17]	35, F	Clear cell	−/−	NR	NR	Total hepatectomy, liver transplantation	Alive
Toriyama et al. [18]	56, M	Clear cell	+/−	2.2	Solitary	Surgical resection, adjuvant chemotherapy	Alive
Kothadia et al. [19]	51, F	Clear cell	−/−	20.7	Multiple	Transarterial chemoembolization	Alive
Chable-Montero et al. [15]	26, F	Follicular thyroid-like	−/−	NR	Solitary	Hepatic resection, adjuvant chemotherapy	Dead
Fornelli et al. [16]	52, M	Follicular thyroid-like	−/−	18	Solitary	None	Alive
Mittal et al. [20]	23, F	Follicular thyroid-like	−/−	12.1	Solitary	Hepatic resection	Alive
Chen et al. [21]	59, F	Follicular thyroid-like	+/−	3	Solitary	Hepatic resection	Alive
Hiraki et al. [22]	45, M	Undifferentiated	+/−	11.5	Solitary	Hepatic resection	Alive
Maeda et al. [23]	56, M	Undifferentiated	−/−	Very small	Multiple	None	Dead

HBV = hepatitis B virus; HCV = hepatitis C virus; M = male; F = female; NR = not reported. EBV = Epstein–Barr virus; cHCC/CCA = combined hepatocellular-cholangiocarcinoma.
